# Nonprofit Hospital Community Benefit in the U.S.: A Scoping Review From 2010 to 2019

**DOI:** 10.3389/fpubh.2020.00072

**Published:** 2020-03-11

**Authors:** Michael D. Rozier

**Affiliations:** Saint Louis University, St. Louis, MO, United States

**Keywords:** hospital community benefit, IRS Form 990 Schedule H, Community Health Needs Assessment (CHNA), community health improvement plan, Tax Exemption

## Abstract

**Background:** U.S. nonprofit hospital community benefit recently underwent significant regulatory revisions. Starting in 2009, the Internal Revenue Service (IRS) required hospitals to submit a new Schedule H that provided greater detail on community benefit activities. In addition, the Affordable Care Act (ACA), which became law in 2010, requires hospitals to conduct community health needs assessments (CHNA) and develop community health implementation plans (CHIP) as a response to priority needs every 3 years. These new requirements have led to greater transparency and accountability and this scoping review considers what has been learned about community benefit from 2010 to 2019.

**Methods:** This review identified peer-reviewed literature published from 2010 to 2019 using three methods. First, an OvidSP MEDLINE search using terms suggested previously by community benefit researchers. Second, a PubMed search using keywords frequently found in community benefit literature. Third, a SCOPUS search of the most frequently cited articles in this topic area. Articles were then selected based on their relevance to the research question. Articles were organized into topic areas using a qualitative strategy similar to axial coding.

**Results:** Literature appeared around several topic areas: governance; CHNA and CHIP process, content, and impact; community programs and their evaluation; spending patterns and spending influences; population health; and policy recommendations. The plurality of literature centered on spending and needs assessments, likely because they can draw upon publicly available data. The vast majority of articles in these areas use spending data from 2009 to 2012 and the first cycle of CHNAs in 2013. Policy recommendations focus on accountability for impact, enhancing collaboration, and incentivizing action in areas other than clinical care.

**Discussion:** There are several areas of community benefit in need of further study. Longitudinal studies on needs assessments and spending patterns would help inform whether organizations have changed and improved operations over time. Governance, program evaluation, and collaboration are some of the consequential areas about which relatively little is known. Gaps in knowledge also exist related to the operational realities that drive community benefit activities. Shaping organizational action and public policy would benefit from additional research in these and other areas.

## Introduction

In 1956, the United States formalized the tax-exempt status for non-profit hospitals. The most prominent aspect of the Internal Revenue Service (IRS) ruling was that hospitals would be tax-exempt if they provided charity care or uncompensated care within their financial ability to do so (1). Less than a decade later, with the passage of Medicare and Medicaid in 1965, there was concern that there would be less need for charity care and tax exemption would no longer be justifiable. Therefore, in 1969 the IRS issued another ruling, which started the conversation about broadening community benefit (2). With this ruling, the IRS established a broader notion of charity, wherein “the promotion of health is considered to be a charitable purpose” and where acceptable activities went beyond charity care as long as the activities were “deemed beneficial to the community as a whole” (3). This ruling granted tax-exempt status to those organizations who met six specific criteria, including: operating an emergency department that cares for anyone regardless of ability to pay; participating in Medicare and Medicaid; creating a governing board that represents the community; and reinvesting surplus funds rather than disseminating them as dividends (3). These 1969 criteria were slightly relaxed in 1983 with a ruling that would remain the primary guidance on the tax-exempt status of hospitals until the mid-2000s (4).

Hospitals' tax-exempt status and the benefit they provide their communities were the subjects of hearings with the Senate Committee on Finance, resulting in a 2008 revision to the IRS code, which now requires hospitals to submit a more detailed accounting of their community benefit activities as part of their tax return (Schedule H, Form 990). The first of these returns were filed in 2009. In 2010, the Patient Protection and Affordable Care Act (ACA) drew additional attention to community benefit in at least three important ways. First, the ACA requires that non-profit hospitals conduct community health needs assessments (CHNA) and develop community health improvement plans (CHIP) to address the most important identified needs at least once every 3 years (5). Most non-profit hospitals in the U.S. conducted their first required CHNA in 2013. Second, the expansion of Medicaid and the inclusion of guaranteed issue creates an environment similar to that following the original passage of Medicare and Medicaid, wherein many question whether tax-exempt status is justified given the possible decrease in charity care (6–10). Finally, the ACA's promotion of population health, primarily through new payment mechanisms, creates a possibility of expanding the notion of community benefit to include social determinants of health. The two reforms—the 2008 IRS ruling and the ACA—are distinct but related. Some results, such as the regular CHNAs, are clearly related to one of those reforms. The 2008 ruling had a fairly narrow focus on stricter reporting guidelines, but the reporting itself could have changed hospitals' behaviors. Therefore, it is difficult to disentangle exactly which evidence is related to each reform.

This study seeks to determine what is known about community benefit since these major federal actions have come into effect. The question for this scoping review is: “What do we know about community benefit in U.S. non-profit hospitals 2010?” The results of this question not only describe the most important areas of knowledge, but also identify those areas with significant gaps.

## Method

Scoping studies have often suffered from lack of consensus on terminologies and methodologies (11). This current study aims “to map the literature on a particular topic or research area and provide an opportunity to identify key concepts, gaps in the research; and types and sources of evidence to inform practice, policymaking, and research” (12). It does this mapping for non-profit hospital community benefit in the United States. One established framework for such work articulates five stages of work: identifying the research question; identifying relevant studies; selecting studies; charting the data; and summarizing and reporting the results (13). The first stage is found in the Introduction. The second and third stages are detailed in this section. The final two stages follow in the Results and Discussion.

This review was largely limited to peer-reviewed articles indexed by academic databases. Some gray literature was also included, particularly editorially-reviewed articles and papers cited by peer-reviewed work. The limitations of this choice are described in the Discussion. The timeframe of interest, 2010–2019, provided one key inclusion criteria for articles.

It can be challenging to identify articles about U.S. non-profit hospital community benefit, as noted by previous literature reviews (14). The challenge is 2-fold. First, the term community benefit is often used more broadly than what is intended in this study. So the search term “community benefit” generates many articles that fall outside the scope of interest. Second, many aspects of community benefit are published without being formally linked to community benefit. For example, articles on non-profit hospital charity care may never note that charity care is a major component of the hospitals' community benefit spending. Therefore, the term “community benefit” is both too broad and too narrow to easily identify articles on the topic.

**Figure 1:**
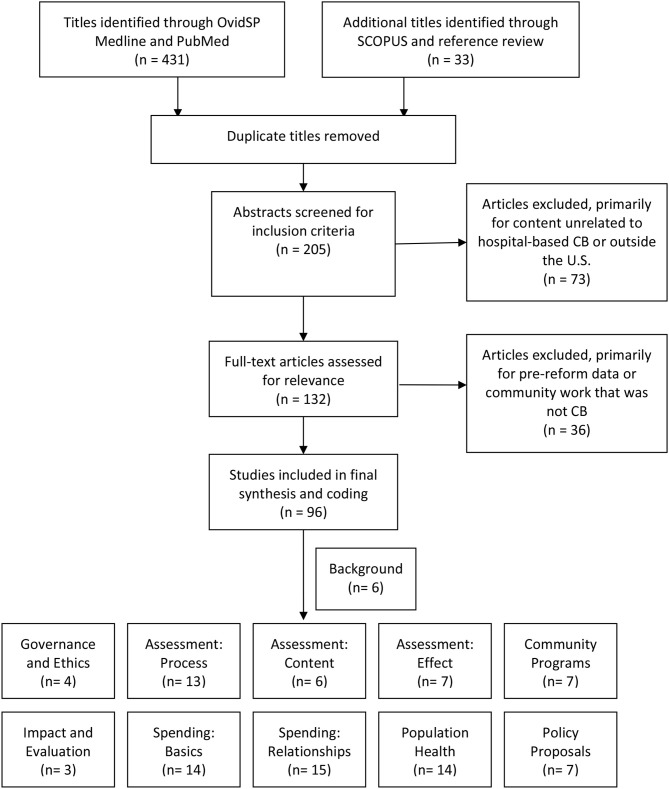
Flow diagram for scoping review.

The search included several strategies (see Figure 1). First, articles were collected using the method previously suggested by community benefit researchers, using the OvidSP MEDLINE search terms (14). Additional articles were collected using PubMed and a search of keywords often associated with community benefit literature. These keywords include: community benefit(s), non-profit hospital(s), charity care, tax-exempt hospitals, tax exempt(ion), community health need(s) assessment(s), schedule H. A final attempt to gather articles was made by conducting a SCOPUS search of the most highly cited articles in community benefit (8, 15, 16).

After reviewing the abstracts of all articles initially identified through the above search strategies, the author eliminated those that did not relate to the study's research question. The most common reason for exclusion was the article addressed hospitals' community health or population health work, but did not connect that work to community benefit. Some articles were excluded if they were published during the accepted date range but exclusively used data that preceded the IRS revision and the ACA. Others were excluded because they addressed community benefit in countries other than the U.S.

All articles were organized using a strategy similar to axial coding of qualitative data (17, 18). The initial topic areas arose from a working group held in 2009 that identified the most salient topics for community benefit scholarship: governance and leadership; ethics; finance and economics; planning, organization, and evaluation; community assessment and engagement; and public policy (19). After sorting into an initial set of topic areas, the articles were further sorted into subtopics. As a scoping review, this study identifies the major topic areas but does not claim to identify all the articles within a given topic area as a systematic review would. At the same time, it does aim to provide the entire map, including those areas that are sparsely populated at the moment.

## Results

Each section of the results begins with the research question that animates the studies in that section (see Table 1). There was also a set of articles that provided descriptive overviews of community benefit, largely explaining the new regulations and requirements. These articles responded to the need for researchers and practitioners to understand the new expectations emerging from the IRS revisions and the ACA. These included some early peer-reviewed overviews (44, 45), with others provided by organizations with interest in educating the public on community benefit, such as from The Hilltop Institute (2, 5) or Catholic Health Association (46, 47). Even several years after the new regulations, perspectives from sources such as *Health Affairs* and *New England Journal of Medicine* described either the current state of affairs for researchers (48) or ways in which new community benefit requirements could shape the provision of health care (49).

**Table 1:** Summary of scoping review topic areas.

	**Key Conclusions Related to Community Benefit**	**Representative Articles**
Governance and ethics	• Boards should feel an ethical obligation in addition to a need for compliance • Requirements related to community health could deepen existing priorities of faith-based organizations	Magill and Prybil (20) McCruden (21)
Community assessment: process	• There is high variation in community-engagement during needs assessments • Vulnerable populations and communities often receive less attention during needs assessments • The process for needs assessments must be context-specific: rural/urban, local health department/not, level of community capacity	Beatty et al. (22) Becker (23) Pennel et al. (16)
Community assessment: content	• Root causes/social factors of community needs are rarely identified • Collaborative needs assessments are often of higher quality • Health equity is often noted as a need but rarely addressed directly by activities	Carroll-Scott et al. (24) Pennel et al. (16)
Community assessment: effect	• Collaboration on needs assessment can have other positive effects, including greater investment in community health activities and increased on-going collaboration • Community members who participate in needs assessments offer unique insights, are more satisfied with the product, and emerge with increased knowledge	Carlton and Singh (25) Franz et al. (26) Kuehnert et al. (27)
Community programs	• Programs typically focus on clinical interventions and address less stigmatized diseases • Aspects of the process that are more highly regulated (needs assessment, reporting) are more consistent across organizations that other aspects (programming, evaluation)	Burke et al. (28) Franz et al. (29) Rozier and Singh (30)
Program impact and evaluation	• Formal evaluation receives little attention at the program level • Community programs may have effects other than at the program level: positive, such as lowering readmissions and negative, such as medicalizing poverty	Chaiyachati et al. (31) Caffrey et al. (32)
Spending and finance: basics	• There is high variation in whether individual hospitals provide more community benefit than they receive in tax exemption, but overall community benefit exceeds hospital tax benefit • ~7.5% of operating expenses go to community benefit, with ~0.4% devoted to community health improvement • Non-profit hospitals generally provide more community assistance than for-profit peers	Bakken and Kindig (33) Rosenbaum (34) Young et al. (15) Valdovinos et al. (35)
Spending and finance: relationships	• Increased state regulation leads hospitals to favor spending on patient care over community health • There are weak, if any, associations between community benefit spending and community characteristics such as higher health needs or level of uninsured • The ACA, especially Medicaid expansion, may be positively associated with higher total community benefit spending; the relationship between bad debt, uncompensated care, and community health improvement is unclear	Begun and Trinh (36) Singh (37) Singh and Young (38) Singh et al. (39) Young et al. (40)
Population health	• The full potential of CB's connection to population health has yet to be realized • The distinction between ‘community building' and ‘community health improvement' is less relevant in an era of population health and social determinants of health • Population health would be more possible with collaboration, shared resources, and common measurement	Begun et al. (41) Corrigan et al. (6) Rosenbaum (34)
Policy recommendations	• Potential improvements include standardizing the CHNA, assessing outcome measures, and requiring more explicit work related to health equity • Policies should better incentivize investment in social determinants and population health	Gruber et al. (42) Rozier et al. (43) Rubin et al. (7)

### Governance and Ethics

“What is the role of hospital leadership related to community benefit?”

With increased public scrutiny, community benefit has taken on new relevance for governing bodies of non-profit health care. Most directly, organizations' tax exemption is dependent upon meeting the new requirements (50, 51). But Magill and Prybil suggested that the need for board oversight goes beyond legal compliance toward an ethical imperative, indicating that board engagement, deliberative communication, and performance measurement are essential markers of meeting the ethical demands of community benefit (20). A 2011 survey found that governing boards had not engaged at this level, with only 36% of systems surveyed having assigned responsibility for community benefit (52). However, this survey was early in the organizational adjustment to new community benefit requirements.

A small number of articles more explicitly addressed matters of ethics and community benefit. McCruden notes that the expectations for deeper community engagement align well with Catholic moral norms and should better orient Catholic hospital toward community health work and reducing health disparities (21). In general, the area of governance and ethics received minimal attention in the literature.

### Community Assessment: Process

“What do we know from studying the process hospitals use to complete CHNAs and CHIPs?”

The new expectations related to CHNAs and CHIPs meant most hospitals had a new skill to learn and researchers had a new process to study. In 2011, the IRS issued a bulletin stating, “a CHNA must take into account input from persons who represent the broad interests of the community served by the hospital facility” (53). At a minimum, this includes, “persons with special knowledge of or expertise in public health; federal, tribal, regional, State, or local health or other departments; … leaders, representatives, or members of the medically underserved, low-income, and minority populations, and populations with chronic disease needs” (53). Most of the existing studies use data from 2012 to 2013, when most hospitals completed their first required CHNA and CHIP.

Several studies from this first cycle identified lessons to be learned in the area of community engagement. Pennel et al. scored 95 CHNAs published in 2013 from Texas hospitals to determine the level of community participation in the process. They found a variety of stakeholders engaged at different phases of the process, but very few hospitals working with a broad spectrum of community members throughout the entire process (54). Using an experience with Yale-New Haven from before the ACA-required CHNA process, Santilli and colleagues suggest strategies such as door-to-door surveys and investing in the workforce needed to carry out community-based work (55). Diaz et al. studied another community-driven prioritization process used by six hospitals in northern California which suggests the importance of integrating qualitative and quantitative data in the process (56).

Two articles ask specifically how the CHNA process can be used to engage vulnerable communities. Lightfoot et al. studied a four-hospital process in Lehigh Valley, PA and found that strategies such as identifying ambassadors from vulnerable communities and encouraging long-term memoranda of agreement were important for success (57). Grant and colleagues studied the CHNA process of Moffitt Cancer Center and concluded that an organization interested in reducing health disparities must engage organizational leaders and community members in an ongoing way and that conversations should be informed by data on demographics and disparities (58). Another did not investigate vulnerable populations but the most vulnerable communities. Singh, Cramer, and Young found that hospitals in communities with the lowest health needs based on County Health Rankings were more likely to have completed CHNA activities than hospitals in communities with the greatest health needs (59). For those interested in community benefit as a mechanism for addressing inequity, this data point could be concerning.

Studies on this topic often employed case-study methodology to describe hospital-community engagement, often in rural settings. For example, Becker looked across multiple examples using the Rural Community Group Model to determine challenges and opportunities for community engagement in rural settings (23). The author found group think to be particularly strong in rural communities where people know each other well (23), which may signal an important risk to be aware of in those instances where strong community health networks exist. Skinner et al. found through interviews representing 21 hospitals in Appalachia that rural hospitals struggle to hire staff for their CHNA process and often lack the resources to address the needs once they are identified (60). Sabin and Levin also provide a case study of a rural hospital meeting community benefit requirements and conclude that collaboration and identifying existing community assets are key to a successful program (61).

Several studies also looked specifically at the collaborative process with local health departments (LHD). In a statewide analysis in Missouri, Beatty, Wilson, and colleagues found significant variation in cooperation and no strong predictors of collaboration between the non-profit hospital and other organizations (22, 62). Laymon et al. provided baseline data from the first CHNA cycle in 2013 and reported that 53% of LHDs collaborated with hospitals on needs assessments, with likelihood increasing in areas of large populations (63). In a case study, Sampson, Gearon and Boe describe a process wherein a hospital-LHD partnership drew upon the local health department's long history of community collaboration and engaged 1,800 Polk County, WI residents in developing the CHNA, many of whom continue to be involved in workgroups to address the identified needs (64).

### Community Assessment: Content

“What do we know from studying the content of CHNAs and CHIPs?”

The content produced by the assessment process provided another focus area of research studies. Most of these studies analyzed content from the 2013 CHNAs and CHIPs and were less likely to be case studies than those that studied process. Several that were case studies or regional analyses often found CHNAs lacking in terms of the depth of collaboration or identifying root causes of community needs. Alfano-Sobsey et al. took in account both process and content as they described the collaboration of organizations in Wake County, NC and their method for prioritizing poverty, access to care, and behavioral health from nine initial areas of concern (65). Akintobi et al. offer another case study, this one with Morehouse School of Medicine Prevention Research Center, describing both the collaborative process as well as the priority areas, including health concerns such as hypertension as well as risk factors such as lack of social cohesion (66). Powell et al. analyzed the content of 15 CHNAs and 10 CHIPs from 2013 produced by Philadelphia-area hospitals. They found little regional coordination between organizations and the implementation strategies generally overlooked behavioral health and social factors, which often arose at top needs (67).

Several studies on content analyzed larger numbers of CHNAs, although most were still from the first cycle of reports. Pennel et al. assessed the quality of 95 Texas hospitals' 2013 CHNAs with 16 criteria, including stakeholder involvement, quality of the data, and clarity of communication. They found those reports done in collaboration with local health departments and those done by consultants were of higher quality (16). Caroll-Scott et al. used data from 2016 CHNAs and CHIPs, and identified a disconnect between CHNAs and CHIPs in the area of health equity. Of the 179 hospitals, 65% of the CHNAs included a term related to health equity, while only 35% of CHIPs did so, and only 9% of the organizations included an activity explicitly promoting health equity (24). Cramer et al. used a much larger sample (*n* = 1,593) to analyze whether organizational or community characteristics were associated with progress toward CHNA implementation (68). One key finding of this analysis is that hospitals reporting high levels of CHNA implementation spent more on community health improvement, which connects the topics of process, content, and effect of CHNAs.

### Community Assessment: Effect

“What, if any, effect does conducting a needs assessment have?”

The CHNA process has the potential to have any number of effects on the organization itself, its collaborators, or the community. One group of studies focused primarily on the effect the process had on collaborating organizations. Carlton and Singh showed that LHD-hospital collaboration on CHNAs was associated with likelihood of coordination on implementation plans and greater hospital investment in community health improvement activities (25). Ainsworth, Diaz, and Schmidtlein found that a four hospital system in northern California approached the CHNA process with broader goals of collaboration in mind and that the effort had several positive effects, including regular meetings after the CHNA process and increased collaboration with other community organizations (69). Analyzing an experience from Lehigh Valley, Matthews, Coyle, and Deegan concluded that broad partnering for CHNAs allowed the group to better identify expertise, helped generate resources for health improvement, and mobilized community partners for the long-term, although the authors did not indicate how they measured these outcomes (70).

Other studies emphasized the effect the CHNA process has on community members. Gold et al. studied a public deliberation with Maimonides Medical Center in Brooklyn, NY and found that the participants emerged with greater knowledge of community health and 95% thought hospitals should use public deliberation to identify priority needs (71). Kuehnert, Graber, and Stone used a web-based survey, generating quantitative measures with Likert scales and qualitative insights from open-ended questions, to discover that those community members who were directly involved in the CHNA process were more satisfied with the final product than those who did not participate (27).

Additional studies assessed whether the CHNA process achieved its primary goal of identifying priority needs and leading to effective strategies to respond to those needs. Through three CHNAs in West Virginia, Bias et al. showed that community participation identified needs that hospital leadership had not identified and that strategies were modified based on the insights provided by community members (72). Additionally, a qualitative study of 21 hospitals in the Appalachian region of Ohio found that hospitals have been formalizing their CHNA processes, cultivating local partnerships, and developing an evidence base for their work (26). Although nearly all of the studies examining the CHNA and CHIP processes are from the first cycle in 2013, there is clearly a good deal of knowledge gained from these initial experiences.

### Community Programs

“What do we know about the programs hospitals support related to community benefit?”

This scoping review did not identify any studies that provided a comprehensive review of the community programs hospitals have supported since the community benefit reforms went into effect. Olden and Hoffman conducted a literature review on hospitals' health promotion services and identified 25 articles, all published before the date range of this scoping review. However, the authors' findings that hospital size and collaborative networks were positively associated with more community programs and that state community benefit laws had no association with programs continue to be relevant. However, the concepts of evaluation or impact were not raised anywhere in the review (73). A literature review by Burke et al. showed that out of 106 programs that met inclusion criteria, over half occurred in the hospital facility and focused on clinical interventions (28).

Some researchers have taken a more targeted approach to the question and analyzed programs in specific areas. For example, Fleischhacker provides a commentary for those in food and nutrition as to how they might leverage hospital resources to increase support for evidence-based programs (74). LeRouge et al. use several hypothetical cases to suggest why telehealth ought to be considered as part of community benefit strategies, namely because telehealth increases access, improves community health, and advances medical knowledge (75). Franz, Skinner, and Kelleher analyzed the 2013 CHNAs at 21 hospitals in Appalachia and conducted interviews to determine why substance abuse was less frequently prioritized compared to obesity and access to care (29). They identified several reasons including lack of resources, risk aversion, and stigma. Rozier et al. took a more theoretical approach to this question and conducted an experiment to determine what factors were most important to non-profit hospital leaders when prioritizing community heath activities. They found the severity of the need and the quality of partnership to be the most important factors for allocation (30).

The topic of community programs is broad and ill-defined, which may partially explain why less literature appears in this section than one might expect. To offer more data as to what is done, Rozier and Singh interviewed 38 directors of community health and mapped the process associated with community health improvement programs, from budgeting to reporting (76). They found consistent and formal processes in areas that are highly regulated, such as assessing needs and reporting dollars spent, but little consistency across organizations in other key areas such as budgeting, allocating resources, or evaluation. Overall, there is less scholarship on the types of community programs associated with community benefit than one might expect. However, this may be because these programs are being shared without being formally identified with community benefit.

### Program Impact and Evaluation

“What effect do community benefit programs have?”

Formal evaluation of community benefit programs received relatively little attention in the literature. Rains et al. use their experience from St. Louis Children's Hospital to provide a methodology for measuring population health impact and showed an increase in process and outcome data for 6 of their 7 community benefit programs (77). It may be that this literature exists but is not readily identifiable as community benefit or it could be that it will take additional cycles of CHNAs and CHIPs for this work to emerge.

In addition to studies about community benefit program evaluation, some have considered other effects that community benefit programs may have on organizations or communities. For example, a recent study analyzed data from 1,405 non-profit hospitals to identify an association between increased community-directed spending and lower preventable readmission rates (31). As another example, Caffrey et al. made an interesting observation that community benefit efforts might have the unintended effect of medicalizing poverty (32). They suggest that an unrepresentative sample of community participants could fail to identify pressing social needs such as employment and violence and that hospitals risk evaluating needs through their expertise of medical care.

### Spending and Finance: The Basics

“How much money do non-profit hospitals spend on community benefit?”

Spending on community benefit constitutes the plurality of literature. This topic area is divided into two sections. The first focuses on those studies that analyze how much is spent and whether that spending changes over time; the second summarizes associations with or influences of spending.

To judge merit of tax exemption, the amount of community benefit spending is often compared to the value of tax exemption, which a national analysis puts at $24.6 billion for the year 2011 (8). Using 2012 data, Herring and colleagues suggest there is variation as to whether individual hospital's community benefit spending exceeds their tax benefit, with 62% providing more than they receive (78). An analysis by Turner et al. drew a similar conclusion with 2010–2012 data from Maryland, finding that hospitals provide more through community benefit than they would provide through taxes (79). However, given that Maryland has its own state-level CB requirements, these results may not hold in other states.

The most frequently cited article on community benefit is an analysis using 2009 data of 1,800 non-profit hospitals from across the country. In it, Young and colleagues found that on average hospitals spent 7.5% of their operating expenses on community benefit, with 0.4% allocated to community health improvement. The variation of total community benefit spending among hospitals was also quite large, ranging from 1.1% of operating expenses for the lowest decile to 20.1% for the highest decile (15). Leider et al. found only a slight increase from 2009 to 2012, with similar distribution across categories (80). An analysis of 2009 data from 127 Wisconsin hospitals found similar results to Young −7.5% of operating expenses were devoted to community benefit, with about 0.4% of operating expenses allocated to community health improvement (81). A study of 53 North Carolina hospitals found a much higher percentage, 14.6% of operating expenses, but this study included Medicare shortfall, which the state allows hospitals to do, but is generally not included in other studies. A state-by-state analysis show significant variation, from 3.8% of operating expenses in North Dakota to 11.9% in Wyoming (33).

One frequent question is whether non-profit hospitals actually provide more community assistance than for-profit hospitals (82). A major challenge with answering such a question is that the two groups are not required to follow the same reporting standards. Two studies, one using national data from 2006 and another using 2011–2013 from California found that non-profit hospitals provided significantly more charity care than their for profit counterparts (35, 83). Another, by Worthy and Anderson, showed that Texas hospitals claiming tax exemption spent more on community services than other hospitals (84). And another study by Coyne and colleagues showed that in Washington state, in 2011, among non-profit hospitals, church-owned hospitals provided more charity care as a percentage of gross revenues than did government or other voluntary hospitals (85). These conclusions align with studies that asked similar questions before the new regulations (86, 87).

The fact that so many studies on community benefit rely on Form 990 Schedule H raises the question as to whether these reports are valid. Rauscher (Singh) and Vyzas compared the self-reported community benefit expenditures from these forms for 218 non-profit California hospitals with other measures of charitable activity. These measures included charity care as reported in financial statements (adjusted with the cost to charge ratio), the Medi-Cal inpatient load, and measures of community orientation and provision of community health services constructed from data in the annual AHA survey. The authors found a strong correlation between the self-reported spending and these other measures, indicating that despite strict standardization, the self-reports are likely an accurate measure of community benefit (88).

### Spending and Finance: Relationships

“What influences how much non-profit hospitals spend?”

Along with knowing how much is spent, other studies have assessed associations with community benefit spending. Although federal laws and regulations are central to community benefit activity, state laws are a possible influence on community benefit spending. One might expect higher spending with state-level laws, but Singh warned about an unintended consequence of minimum thresholds actually lowering spending, in an article where she suggested that spending be just one component of assessing an organization's claim for tax exemption (89). The results on state influence are mixed. Singh et al. using 2009–2011 data, showed that hospitals often respond to increased regulation by favoring spending on patient care over community health improvement (39). According to an early study by Begun and Trinh, states with additional laws related to community benefit spending, hospitals spent less on community health improvement (36). Yet Johnson et al. found state laws increased total community benefit spending, but that rural hospitals responded to state community benefit laws to a lesser degree than did urban hospitals (90).

Another possible influence on patterns of spending is community characteristics, especially level of community health need. In a national analysis, Singh et al. created standardized measures of county health needs using the 2010 County Health Rankings and found that overall community benefit was higher for hospitals in counties with higher health needs, but that spending on community health improvement was not (91). This raises a question of whether there may be trade-offs between spending on charity care and other spending such as community health improvement. In an analysis of Maryland hospitals from 2006 to 2010, Singh found that there was no evidence of such a trade-off. Despite the fact that hospitals in the poorest areas of the state bear a larger burden of uninsured patients, they did not show evidence of such a trade-off. Moreover, a trade-off was not seen during the 2008 recession, wherein one may expect to see a reduction in spending on community health programs to compensate for the increase in charity care (37). Given that Maryland has state-level CB requirements and a Medicare waiver that standardizes reimbursement across all payers, the results may not be entirely generalizable. There was also no relationship between governmental public health spending and community benefit spending on community health improvement (38, 92). Beahr et al. also found no association between community need and per capita community benefit expenditures (93). Another study by Chaiyachti, Qi, and Wener found neither total community benefit spending nor community-directed contributions varied based on community characteristics such as percentage of uninsured and education levels (94).

Several recent studies have examined whether the ACA influenced the amount or patterns of spending on community benefit. This question was largely precipitated by the idea that increased insurance coverage would lessen the need for uncompensated care (95) and create the possibility of increased spending on community health improvement (96). Nikpay, Buchmueller, and Levy found that early Medicaid expansion in Connecticut resulted in more Medicaid discharges, but no change in uncompensated care (9). The only nationwide study of the ACA's effect on community benefit spending found a modest increase in total spending, from 7.6 to 8.1% of operating expenses, from 2010 to 2014, but no effect on community health spending (40). And yet, in an analysis of just teaching hospitals, Alberti, Sutton, and Baker found between 2012 and 2015, charity care decreased by 16.7% but total spending increased 20.1% (97). Those in Medicaid expansion states increased spending on Medicaid shortfall and subsidized health services more quickly than non-expansion states.

Finally, in a time of ever-shrinking operating margins and greater dependence on non-operating incomes, Song, McCullough, and Reiter show that non-operating income does not influence total spending on community benefit (98).

### Population Health

“How does community benefit interact with the increased attention to population health and population health management?”

Although many of the articles in this topic area could be included either with evaluating impact or with policy recommendations, the large number of articles specifically relating to population health warranted its own section. Two empirical articles illustrate early skepticism on community benefit and population health. Pennel et al. assessment of 2013 CHNA content and interviews with stakeholders led them to believe that non-profit hospital involvement in population health was unlikely (99). Along the same lines, after analyzing 23 organizations' CHIPs and finding that very few were addressing the structural causes of health inequity, Begun et al. proposed a 5-point scale to help organizations focus on higher-impact, population health activities (41). But other articles, some from advocacy organizations, continue to suggest that non-profit hospitals should take a larger role in population health improvement and to use community benefit as cornerstone of such work.

The Democracy Collaborative (100), Catholic Health Association (101), Community Catalyst (102), and the then-Institute of Medicine (103) are just some of the organizations to note community benefit's potential in advancing population health. Sara Rosenbaum suggested that the definition of community benefit be expanded to include community building activities (34, 104) and that the IRS be more directive in hospitals reallocating resources for community health improvement (104). Bakken and Kindig did projections to show that community health spending would increase 3-fold if hospitals were required to spend a certain percentage of community benefit dollars on community health improvement (10% minimum, which would increase as hospital profitability increased) (105). But this kind of shift does not just happen, which is why other articles, often appearing in gray literature, offer suggestions as to how an organization can best manage community benefit's relationship with population health.

Early on, one industry publication noted the need to use evidence-based interventions in order to maximize effectiveness of community benefit programs (106). Another early article noted that if an organization wanted to commit to population health, it would need to develop a comprehensive strategy that addressed cultural and structural, including adopting community well-ness as a strategic priority (107). Corrigan, Fischer, and Heiser also wrote about strategy, but focused on the need for regional collaboration, shared resources, and common measurements (6). Several organizations have also built tools to help organizations in this effort. For example, Community Catalyst developed a dashboard tool to assess a community benefit program's commitment to equity and engagement (108) and Health Resources in Action created a tool for strategic planning and cultural alignment (109).

### Policy Recommendations

“What changes to policy could improve community benefit?”

With the major changes to community benefit in 2009 and 2010, increased attention and experience with the new requirements have created an opportunity for scholars and practitioners to offer suggestions as to how community benefit-related policy might be further modified. An early summary of policy proposals suggested three categories: transparency, accountability, and population health (7). This scoping review found leveraging community benefit for population health to be the most frequent policy recommendation. Several suggested this should be done by modifying the CHNA. Crossley suggested that better alignment with community health could arise with more transparent and accountable guidance related to CHNAs (110), with Gruber and colleagues going even further to suggest a standardized CHNA format would increase accountability for health outcomes (42). Rubin, Singh, and Young suggested that assessing outcomes such as community-level health measures would be a better approach than assessing inputs such as CHNAs or spending (111). Other authors have suggested that population health goals would benefit from clarifying the ‘community building' category so that non-profit hospitals are better incentivized to invest in the social determinants of health (43, 112).

In addition to population health, several articles recommended policies that facilitate better coordination of community benefit efforts. Some have noted that non-profit hospitals and local public health agencies could coordinate their efforts if their compliance activities, such as needs assessments, were better aligned (43, 113). Rozier, Goold, and Singh, who offered better coordination with public health as one of four policy proposals, also suggested that health equity be made an explicit expectation of community benefit activities (43). Rubin, Singh, and Jacobson make a case for greater accountability, specifically suggesting that the IRS assess population-health performance measures which are already included in the required CHIPs (111).

## Discussion

This scoping review identified a significant amount of literature published on community benefit since the IRS revisions and ACA regulations, in all areas of scholarship identified by a 2009 working group (19). We know, for example, that overall spending is significantly greater than the amount that would be paid in taxes, although that is certainly not true for every individual hospital. We also know that spending as a percentage of operating expenses has remained relatively steady over the early years following new regulations and that there are very few factors, either organizational, or community, that are associated with amount or distribution of spending. Several proposed policies, however, are aimed at making it more likely that community-level factors, particularly community health needs, are more strongly associated with spending and spending patterns. Such an association would also make it more likely that community benefit activities better contribute to addressing health inequities.

In addition to standardized reporting on spending, the new regulations have a strong focus on increasing collaboration with communities and encouraging greater rigor in distribution of resources. Most studies in these areas use CHNAs, CHIPs, and the American Hospital Association's annual survey from 2013 or earlier. When studies using data from 2014 and beyond are more common, we will better understand whether there has been meaningful progress in quality of collaboration or selection of interventions. From the early years, we know that collaboration was quite uneven and that social determinants of health were not a central focus of community benefit efforts. Studies that evaluate second and third cycles of needs assessments and implementation strategies will hopefully illuminate whether collaboration has improved and whether there is greater rigor is program selection and program evaluation. It is also possible that studies in the coming years will consider whether there are meaningful improvements to community health indicators because of community benefit efforts, either from direct investment or from community benefit helping create community ecosystems that are more attuned to community health.

Many have suggested that the ACA would lead to new opportunities related to community benefit spending, namely a shift away from uncompensated care toward community health investment. Despite significant attention being paid to population health and headlines about hospitals and health systems investing in population health, we do not yet have meaningful data showing that health care organizations have been able to shift their spending away from clinical care toward community health and community building. This may still be in the offing, or it could be that most of uncompensated care for the uninsured simply shifted to shortfall from Medicaid and other means-tested programs. We must also consider that any changes, either in spending or other elements that rely on organizational support, may in part be a result of organizations developing better procedures for recording spending or communicating activity.

There is a reason that many of the articles in this review are proposing new regulations related to community benefit. It seems that organizations have responded to the expectations set forth in the IRS revisions and ACA regulations, but that the changes had submerged goals such as improving health equity or shifting toward social determinants of health, that do not appear in the regulations. In reviewing the relationship between regulations and organizational behavior, it is often difficult to tell the degree to which organizations go beyond the minimum of what is required. For example, we know that boards are approving CHNAs and CHIPs, but we do not know how engaged governance structures are beyond the approval process. And we know that collaboration occurs during the needs assessment process and that the depth of collaboration is highly variable, but it is not clear how regulations could be amended to make meaningful collaboration during needs assessment and other stages of the community benefit process more likely. In part, some of the concerns that lead to new policy proposals could be ameliorated with better data on community benefit activities. However, other concerns will likely need to be addressed with new policy.

### Gaps in Knowledge

This review shows there are still many areas of community benefit in need of further study. For those areas where public information is more easily available, namely in the forms of CHNAs, CHIPs, and Schedule H Form 990s, most of the current information come from the early years of this new community benefit activity. Studying changes in CHNAs and CHIPs over time will be more possible now that most hospitals have completed three full cycles. At the time of this review, there were no studies that took account of the 2019 CHNA cycle. Subsequent studies will hopefully do so. While there have been some studies that considered longitudinal aspects of spending, the significant changes to the U.S. health care system from 2012 and the greater openness of the IRS to include housing and other determinants of health as community benefit (114) offer additional opportunities to study patterns of and associations with community benefit spending. If community benefit spending truly remained consistent from 2010 to 2019, it would be about the only element of U.S. health care spending that did not meaningfully change during that time.

In addition, there are significant gaps in knowledge in the topic areas for which there is no standardized, publicly available data. Governance, resource allocation processes, and program evaluation are just some of the areas we know very little about. In general, internal operations related to community benefit largely remain a “black box.” If we do no know about these areas, the information rarely extends beyond a case study. While case studies certainly have their place in building a base of knowledge, it would also be beneficial to have larger datasets in these areas that would allow for more generalizable conclusions. If health care organizations are interested in broadening the community benefit conversation beyond spending, as I suspect many are, it would behoove them to help researchers more easily secure the data necessary to answer questions associated with these other areas of community benefit.

### Limitations

There are two major limitations to this scoping review worth noting. First, it was largely restricted to peer-reviewed literature. Many advocacy groups and industry associations have produced important work related to community benefit and only some of them have been cited in this review. The focus on peer-reviewed literature may have resulted in some topic areas not being as robustly represented as they could be, especially related to governance and policy recommendations. Second, some areas of research may be less likely to use traditional keywords associated with community benefit. For example, community programs and program evaluation that were, in fact, part of a hospital's community benefit portfolio, may never indicate that they were associated with the hospital's community benefit efforts. Therefore, this review may underrepresent the literature, particularly related to these topic areas. Finally, some may wish that this scoping review was a systematic review or that it accounted for the literature published before 2010, but this study maps the literature of an important moment in community benefit and provides areas where additional work can and should be done.

## Conclusion

Most people in places where there is a non-profit hospital are likely interested in one question related to community benefit. “Are we better off because we have a non-profit hospital in our community?” From a strictly financial perspective, the answer is usually “yes.” The research above shows that most non-profit hospitals contribute more back to the community than they would pay in taxes. However, if pushed, I suspect most people would want more. Most people would ultimately want to know, “Are we using the resources non-profit hospitals provide back to the community as wisely as possible?” The answer to that question is less certain. In order to get to yes, organizations will need to overcome organizational inertia to better identify root causes of illness, choose more effective programs, create stronger partnerships, do more rigorous evaluations, and much more. In order for these to occur, we need more research and perhaps new policies to shape behavior. Ten years after some major reforms to community benefit, we have learned many lessons in building healthier communities. Nevertheless, we have also learned there is a much to be done before the potential is fully met.

## Author Contributions

MR designed the study, conducted the analysis, and wrote the manuscript.

### Conflict of Interest

The author declares that the research was conducted in the absence of any commercial or financial relationships that could be construed as a potential conflict of interest.
